# Viral Acute Lower Respiratory Tract Infections (ALRI) in Rural Bangladeshi Children Prior to the COVID‐19 Pandemic

**DOI:** 10.1111/irv.70062

**Published:** 2024-12-19

**Authors:** Megan E. Reller, Kayur Mehta, Eric D. McCollum, Salahuddin Ahmed, Jack Anderson, Arunangshu D. Roy, Nabidul Haque Chowdhury, Samir Saha, Lawrence H. Moulton, Mathuram Santosham, Abdullah H. Baqui

**Affiliations:** ^1^ Department of Medicine Duke University School of Medicine Durham North Carolina USA; ^2^ Duke Global Health Institute Durham North Carolina USA; ^3^ Durham Veterans Affairs Medical Center Durham North Carolina USA; ^4^ Department of International Health Johns Hopkins Bloomberg School of Public Health Baltimore Maryland USA; ^5^ Global Program in Respiratory Sciences, Department of Pediatrics, Eudowood Division of Pediatric Respiratory Sciences Johns Hopkins University School of Medicine Baltimore Maryland USA; ^6^ Projahnmo Research Foundation Dhaka Bangladesh; ^7^ Child Health Research Foundation Dhaka Bangladesh

**Keywords:** ALRI, Bangladesh, child, infant, viral

## Abstract

**Background:**

Acute lower respiratory tract infections (ALRIs) remain the leading infectious cause of death among children < 5 years, with viruses contributing to a large proportion of cases. Little is known about the epidemiology and etiology of viral ALRI in rural Bangladesh.

**Methods:**

We enrolled 3‐ to 23‐month‐old children with ALRIs attending a subdistrict hospital outpatient clinic in Sylhet district in Bangladesh. Trained study physicians ascertained the cases and obtained nasopharyngeal swabs to detect 19 respiratory viruses by multiplex PCR using the Luminex Integrated System NxTAG Respiratory pathogen panel.

**Results:**

Between August 2016 and September 2017, we enrolled 1477 children. Median age was 10 months; 58.1% were male. Forty‐seven percent presented during autumn (mid‐June to mid‐October). About a third had temperature ≥ 101°F, 95.4% had cough in the previous 3 days, 72.0% had fast breathing, and 80.0% had chest indrawing. Alveolar consolidation occurred in 23.9%, and 4.4% were hypoxemic (saturation < 90% on room air). Nineteen percent required hospitalization; 79.1% of them were discharged within 48 h. A respiratory virus was identified in 81.8%, majority (75.8%) with single virus isolation. Rhinoenterovirus was most commonly identified (HRV/HEV, 37.9%), followed by respiratory syncytial virus (RSV, 20.2%) and human metapneumovirus (hMPV, 11.7%). Rhinoenterovirus was detected year‐round; RSV was detected during August–November and hMPV during December–March.

**Conclusions:**

Respiratory viruses were identified in a majority (82%) of children under 2 years of age presenting with ALRI in rural hospitals of Bangladesh. These findings have implications for future study and potentially for surveillance, antimicrobial stewardship, vaccine program planning, and policy.

## 1 Background

Acute lower respiratory infections (ALRIs) are a leading cause of morbidity and mortality in children < 5 years of age globally. [1]. ALRIs are responsible for almost 20% of all deaths of children aged less than 5 years worldwide [2] and are responsible for up to 30% of pediatric admissions in low‐ and middle‐income countries (LMICs) [3].

Despite a 65% decline in under‐5 mortality between 1990 and 2015 [4], Bangladesh is still one of five countries in the world accounting for > 50% of global ALRI cases in children < 5 years. It has one of the highest population densities in the world (~1300 people/km^2^) [5], a risk factor for ALRI transmission, and prevalence of childhood malnutrition, a risk factor for mortality [6]. Population‐based studies in rural Bangladesh by our group previously found an ALRI incidence of 230 per 1000 child‐years in children < 5 years [7] and documented ALRI‐related hospital admission rates of 50.2–53.6 and 101.1–145 per 1000 child‐years for those < 5 and < 1 year of age, respectively [7, 8]. Another study showed that ALRIs caused 39% of pediatric hospitalizations and 40%–60% of total pediatric outpatient department visits in Bangladesh [9].

Evidence‐based public health approaches to prevent ALRI require rigorous studies to define the epidemiology and etiology of the disease. Multiple viruses, including influenza virus, respiratory syncytial virus (RSV), human metapneumovirus (hMPV), parainfluenza virus (PIV), human rhinoenterovirus (HRV/HEV), and human coronavirus (HCoV), have been implicated as etiologies of ALRI in children. The seminal PERCH study implicated multiple viruses as major causes of ALRI globally, including urban Bangladeshi children < 5 years, and found that respiratory viruses have heterogeneous circulation patterns [10]. However, data from rural settings are lacking. In this study, we sought to delineate the viral etiology and epidemiological and clinical characteristics of viral ALRI in children < 2 years of age in the rural Sylhet region of Bangladesh.

## 2 Methods

### 2.1 Study Site

Between 2014 and 2018, the Projahnmo Study group, a partnership of Johns Hopkins University (USA) with the Ministry of Health and Family Welfare (MoHFW) of the Government of Bangladesh, and Bangladeshi nongovernmental organizations conducted an assessment of national introduction of pneumococcal conjugate vaccine in Bangladesh. The study area, population, and surveillance methods were previously described [11]. In short, the study was conducted in three subdistricts (Zakiganj, Kanaighat, and Beanibazar) of Sylhet district, which have a population of ~770,000 yielding an annual birth cohort of ~20,000. Projahnmo has extensive research infrastructure, including Global Positioning System mapping, a complete census and background characteristics of the entire population, mechanisms for community‐based sampling, case identification, referral, specimen collection, transport, state‐of‐the‐art laboratories, and a data center. It has established surveillance in three subdistrict hospitals, which are staffed by trained study physicians. We used these hospitals for case detection, specimen collection, and transport. The current study was nested within the parent study described above.

### 2.2 Study Population

As part of the parent study [11, 12, 13], we established community surveillance for ALRI and ALRI. The study population for this study was children aged 3–23 months. Trained community health workers (CHWs) visited all households once every 2 months to teach mothers/caregivers how to recognize signs and symptoms of ALRI and asked them to take their sick children to one of the study‐designated health facilities. They also collected data on reported signs/symptoms of ALRI and care seeking.

### 2.3 Surveillance, Case Definition, and Study Procedures

Because respiratory viral pathogens predominate in children < 2 years of age, we screened 3–23 months old children attending the study hospitals for enrollment. Study physicians at the subdistrict hospitals were trained to identify children with clinical signs and symptoms of ALRI.

ALRI was defined as lower chest indrawing or cough or difficulty breathing and either fast breathing (respiratory rate ≥ 50 breaths/min for infants aged 3–11 months or ≥ 40 breaths/min for 12‐ to 23‐month‐olds) or another clinical sign of severe illness (persistent nasal flaring, cyanosis, head nodding or tracheal tugging, grunting, stridor while calm, hypoxemia, decision to hospitalize/refer, or WHO danger sign) [11, 14].

Children were excluded if they were previously enrolled within 30 days to avoid misclassification of a prolonged episode as a new episode. After obtaining written informed consent, demographic and clinical data, along with a nasopharyngeal swab, were collected by study physicians after consent procedures. In addition, chest radiography was performed using an analog unit (POLYMOBIL® Plus, Siemens, Erlangen, Germany) and CR Fuji Film cassette reader to digitize images. Weight was measured using standardized Tanita scales, and height or infant length (as appropriate) were measured using locally available scales by clinical assistants at the study hospitals. Outcome data were collected for children admitted to hospital.

### 2.4 Respiratory Pathogen Molecular Testing

Nasopharyngeal specimens collected with neonatal flocked swabs (FLOQSwabs(R)) were placed into universal transport medium tubes (UTM(R)) (Copan Italia SpA, Brescia, Italy) and stored at −80°C within 2 h of receipt. Specimens were shipped to Duke University, Durham, NC, USA, on dry ice where real‐time reverse transcription–polymerase chain reaction (PCR) with the Luminex Integrated System NxTAG Respiratory Pathogen Panel platform was performed. The Luminex platform detects 19 respiratory viruses (RSV A and B; nonspecific influenza A; influenza A subtypes H1, H3, and 2009 H1N1; influenza B; parainfluenza 1–4; human metapneumovirus (hMPV); adenovirus; human rhinoenterovirus (HRV/HEV); coronavirus types NL63, HKU1, 229E, and OC43; and human bocavirus) and three bacteria ( *Chlamydophila pneumoniae* , *Legionella pneumophila* , and *Mycoplasma pneumoniae* ) [15].

### 2.5 Statistical Analyses

Data related to baseline demographics and clinical presentation were analyzed using descriptive statistics, expressed as frequencies and proportions. Descriptive statistics were also used to describe the distribution of viruses identified and the seasonality, and the clinical characteristics of various viral infections. For seasonality, we used the classification used by Stevens et al. [16], to define summer as the period between April 15 and June 14; monsoon, June 15 and August 14; autumn, August 15 and October 14; late autumn, October 15 and December 14; winter, December 15 and February 14; and spring, February 15 and April 14. Per the WHO, we defined overcrowding as more than three persons per habitable room [17]. Chest radiographs were obtained and interpretated by a reading panel of eight Bangladeshi radiologists and pediatricians trained and calibrated to interpret images according to WHO chest radiograph methods for vaccine studies [18]. Stunting, wasting, and underweight were defined as length‐for‐age, weight‐for‐height, and weight‐for‐age *z* scores below −2 standard deviations (SD) from the median of the WHO child growth standards, respectively [19].

### 2.6 Ethics

The National Research Ethics Committee of Bangladesh Medical Research Council and the institutional review boards of the Johns Hopkins and Duke Schools of Medicine reviewed and approved the study's protocol.

## 3 Results

### 3.1 Baseline Demographics

**FIGURE 1:**
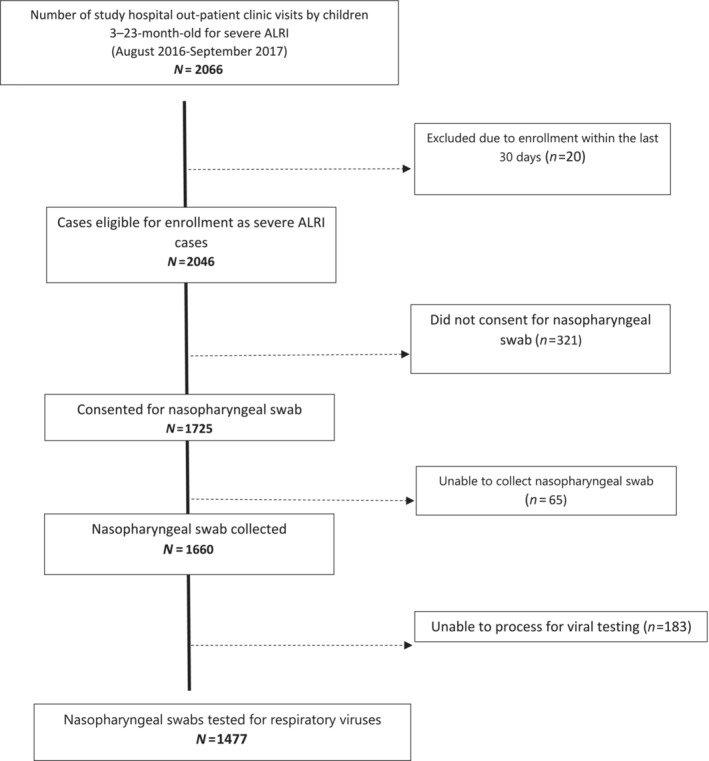
Study profile of children 3–23 months enrolled with ALRI tested for respiratory viruses, rural Bangladesh, 2016–2017.

Between August 2016 and September 2017, 2066 hospital outpatient visits were recorded for ALRI in children aged 3–23 months from the study area. Of these, 1477 (71.5%) had a nasopharyngeal specimen tested for respiratory viruses (Figure 1). Baseline demographics and clinical features of ALRI in children tested for respiratory viruses are shown in Tables 1 and 2. The median age of children enrolled was 10 months, and 58.9% (871/1477) were male. Most of the children presented with ALRI during the autumn season (mid‐June to mid‐October). Overcrowding was noted in three‐fourths of the households, and 71.7% (1060/1477) of these children were exposed to cigarette smoking (passively). A third of the children whose swabs were tested for respiratory viruses were either stunted, wasted, or underweight. The immunization coverage was high, with 93.7% (1383/1477) of the children having received at least one dose of the DPT/Pentavalent vaccine (Table 1).

**TABLE 1:** Characteristics of children enrolled with ALRI and tested for respiratory viruses.

Characteristics^a^	Total (*n* = 1477)
Age in months (median, IQR)	10 (6–15)
**Age in months (categorical)**	
3–5 months	329 (22.2)
6–11 months	572 (38.7)
12–17 months	335 (22.6)
18–23 months	241 (16.3)
**Gender, male**	871 (58.9)
**Season of presentation** ^b^	
Summer	80 (5.4)
Monsoon	302 (20.4)
Autumn	493 (33.3)
Late autumn	238 (14.1)
Winter	308 (18.3)
Spring	56 (3.7)
**Crowding status**	
≤ 3 people/room	835 (56.5)
> 3 people/room	642 (43.5)
**Household wealth tertiles**	
Upper	468 (31.7)
Middle	447 (32.3)
Lower	472 (34.0)
**Maternal education**	
No education	205 (13.8)
Primary (1–5)	582 (39.4)
Secondary (6–10)	641 (43.4)
Higher secondary and above (11+)	49 (3.3)
**Paternal education**	
No education	421 (28.5)
Primary (1–5)	619 (41.9)
Secondary (6–10)	350 (23.7)
Higher secondary and above (11+)	87 (5.9)
**Nutritional status of the child**	
Underweight	511 (34.6)
Stunted	502 (33.9)
Wasted	521 (35.2)
**Exposure to smoking** ^c^	1060 (71.7)
**PCV vaccine status** ^a^	
No dose	111 (7.5)
1 dose	140 (9.4)
2 doses	318 (21.5)
3 doses	908 (61.4)
**DPT3/Pentavalent3 vaccine status** ^a^	
No dose	94 (6.3)
1 dose	141 (9.5)
2 doses	177 (11.9)
3 doses	1065 (72.1)

^a^ Percentages are all column percentages, unless indicated otherwise. Percentages are rounded off to the first decimal point.

^b^ Summer = April 15–June 14; monsoon = June 15–August 14; autumn = August 15–October 14; late autumn = October 15–December 14; winter = December 15–February 14; spring = February 15–April 14 (15).

^c^ Refers to passive cigarette smoke exposure.

**TABLE 2:** Clinical features of children presenting with ALRI.

Characteristics^a^	Total *n* = 1477	3–5 months *n* = 329	6–11 months *n* = 572	12–17 months *n* = 335	18–23 months *n* = 241
**Reported symptoms (last 3 days)**					
Cough	1410 (95.4)	316 (96.0)	550 (96.1)	317 (94.6)	227 (94.1)
Difficulty breathing	970 (65.6)	227 (68.9)	379 (66.2)	216 (64.4)	148 (61.4)
Runny nose	595 (40.2)	130 (39.5)	225 (39.3)	141 (42.0)	99 (41.0)
Reported fever	1107 (74.9)	225 (68.3)	441 (77.0)	265 (79.1)	176 (73.0)
**Physical examination**					
**Temperature**					
≥ 101°F	566 (38.3)	91 (27.6)	224 (39.1)	152 (45.3)	99 (41.0)
99.5°F–100.9°F	216 (14.6)	42 (1.2)	89 (15.5)	49 (14.6)	36 (14.9)
98.6°F–99.4°F	294 (19.9)	77 (23.4)	111 (19.4)	63 (18.8)	43 (17.8)
Afebrile	401 (27.1)	119 (36.1)	148 (25.8)	71 (21.1)	63 (26.1)
Observed cough	438 (29.6)	107 (32.5)	160 (27.9)	102 (30.4)	69 (28.6)
Fast breathing	1064 (72.0)	242 (73.5)	367 (64.1)	257 (76.7)	198 (82.1)
Chest in‐drawing	1183 (80.0)	279 (84.8)	454 (79.3)	263 (78.5)	187 (77.5)
Nasal flaring	129 (8.7)	25 (7.5)	52 (9.0)	26 (7.7)	26 (10.7)
Grunting	6 (0.0)	1 (0.0)	2 (0.0)	2 (0.0)	1 (0.0)
Head nodding or tracheal tugging	171 (11.5)	40 (12.1)	73 (12.7)	32 (9.5)	26 (10.7)
**Oxygen saturation**					
94–100	1154 (78.1)	260 (79.0)	437 (76.3)	261 (77.9)	196 (8.1)
90–93	110 (7.4)	30 (9.1)	49 (8.5)	16 (4.7)	15 (6.2)
< 90	66 (4.4)	22 (6.6)	23 (4.0)	15 (4.4)	6 (2.5)
Missing	147 (9.9)	17 (5.1)	63 (11.0)	43 (12.8)	24 (9.9)
**Lung auscultation**					
Crepitations	1014 (68.6)	228 (69.3)	405 (70.8)	223 (66.5)	158 (65.5)
Wheeze on auscultation	426 (28.8)	102 (31.0)	165 (28.8)	84 (25.0)	75 (31.1)
**General danger sign**					
Convulsions	42 (2.8)	1 (0.0)	11 (1.9)	19 (5.6)	11 (4.5)
Lethargy	4 (0.0)	0 (0.0)	1 (0.0)	3 (0.0)	0 (0.0)
Unable to eat or drink	0 (0.0)	0 (0.0)	0 (0.0)	0 (0.0)	0 (0.0)
Vomits everything	1 (0.0)	1 (0.0)	0 (0.0)	0 (0.0)	0 (0.0)
**Chest radiography**					
Alveolar consolidation only	53 (3.5)	13 (3.9)	20 (3.4)	11 (3.2)	9 (3.7)
Other infiltrate only	249 (16.8)	68 (20.6)	95 (16.6)	57 (17.0)	29 (12.0)
Alveolar consolidation and other infiltrate	302 (20.4)	81 (24.6)	115 (20.1)	68 (20.2)	38 (15.7)
**Admitted to hospital**	292 (19.7)	84 (25.5)	122 (21.3)	60 (17.9)	26 (10.7)
**Duration of hospitalization** ^b^					
< 1 day	24 (8.2)	6 (7.1)	10 (8.1)	5 (8.3)	3 (11.5)
1–2 days	207 (70.8)	56 (66.6)	89 (72.9)	41 (68.3)	21 (80.7)
3–4 days	51 (17.5)	18 (21.4)	18 (14.7)	13 (21.6)	2 (7.6)
≥ 5 days	10 (3.5)	4 (4.7)	5 (4.0)	1 (1.6)	0 (0.0)
**Outcome of hospitalization** ^b^					
Cured and discharged	284 (97.2)	82 (97.6)	118 (20.6)	60 (96.7)	24 (92.3)
Referred	4 (1.4)	1 (1.2)	3 (2.4)	0 (0.0)	0 (0.0)
Left against medical advice	3 (1.0)	0 (0.0)	1 (0.8)	0 (0.0)	2 (7.7)
Died	1 (0.4)	1 (1.2)	0 (0.0)	0 (0.0)	0 (0.0)

^a^ Percentages are all column percentages, unless indicated otherwise. All percentages are rounded off to the first decimal point.

^b^ Among 292 children who required hospitalization.

### 3.2 Clinical Signs and Symptoms

Over one‐third (566/1477) of the children presented with temperature ≥ 101°F; older children were more often noted to have this finding. Ninety‐five percent (1410/1477) of the children's caregivers reported cough in the last 3 days, 29.6% (438/1477) had cough observed by the study clinicians, 72% (1064/1477) children had fast breathing, and 80% (1183/1477) had chest indrawing. Crepitations and wheezing were noted during lung auscultation in 68.6% (1014/1477) and 28.8% (426/1477) children, respectively. On chest radiography, alveolar consolidation with or without other infiltrates were noted in 23.9% (355/1477) of the children. Hypoxemia (SpO_2_ < 90%) was documented in 4.9% (66/1330) of the children with available oxygen saturation readings. Nineteen percent (292/1477) of the children required hospitalization, and the remaining cases were treated as outpatients. Of the hospitalized children, 79% (207/292) were discharged within 48 h of admission. One child died, and three children left against medical advice during hospitalization (Table 2).

### 3.3 Viral Etiology Identified, Seasonality, and Clinical Presentation Among Those With Specific Viruses

A respiratory virus was identified in 81.8% of children, with a majority (75.8%, 917/1209) having only one virus identified. Among those with more than one virus identified, most (85.2%, 249/292) were dual infections (Table 3).

**TABLE 3:** Respiratory viruses identified among 1477 cases with ALRI.

Virus	Total positive	Single infection^a^	Multiple infection^a^	Infection with 2 viruses^b^	Infection with ≥ 3 viruses^b^
Any^c^	1209 (81.8)	917 (75.8)	292 (24.2)	249 (85.2)	43 (14.8)
HRV/HEV	560 (37.9)	362 (64.6)	198 (35.4)	165 (83.3)	33 (16.7)
RSV A^d^	111 (7.5)	77 (69.3)	34 (30.7)	31 (91.1)	3 (8.9)
RSV B^d^	188 (12.7)	141 (75)	47 (25)	41 (87.2)	6 (12.8)
HMPV	173 (11.7)	113 (65.3)	60 (34.7)	44 (73.3)	16 (26.7)
Influenza A^e^	66 (4.4)	51 (77.2)	15 (22.8)	9 (60)	6 (40)
Influenza B^e^	26 (1.7)	16 (61.5)	10 (38.5)	8 (80)	2 (20)
Adenovirus	85 (5.7)	28 (32.9)	57 (67.1)	44 (77.1)	13 (22.9)
PIV 1	34 (2.3)	19 (55.9)	15 (44.1)	10 (66.6)	5 (33.4)
PIV 2	2 (0.1)	1 (50)	1 (50)	1 (100)	0 (0)
PIV 3	42 (2.8)	24 (57.1)	18 (42.9)	15 (83.3)	3 (16.7)
PIV 4	18 (1.2)	14 (77.7)	4 (22.3)	3 (75)	1 (25)
HCoV 229E	10 (0.6)	5 (50)	5 (50)	2 (40)	3 (60)
HCoV NL63	17 (1.1)	8 (47)	9 (53)	7 (77.7)	2 (22.3)
HCoV OC43	24 (1.6)	12 (50)	12 (50)	7 (58.3)	5 (41.7)
HCoV HKU1	5 (0.3)	1 (20)	4 (80)	3 (75)	1 (25)
Bocavirus	163 (11.0)	37 (22.6)	126 (77.4)	99 (78.5)	27 (21.5)
*Chlamydophila pneumoniae*	19 (1.2)	7 (36.8)	12 (63.2)	6 (50)	6 (50)
*Legionella pneumoniae*	0 (0)	0 (0)	0 (0)	0 (0)	0 (0)
*Mycoplasma pneumoniae*	4 (0.2)	1 (25)	3 (75)	3 (100)	0 (0)

^a^ Proportion in parenthesis indicates proportion of single/multiple infection cases of total positive (i.e., row percentage).

^b^ Proportion in parenthesis indicates proportion of those with infection with 2/≥ 3 viruses of those with multiple infection.

^c^ 1209/1477 subjects enrolled in the study with ALRI were found to have fewer than one virus.

^d^ Taken together; 299/1477 (20.2%) subjects had RSV.

^e^ Taken together; 92/1477 (6.1%) subjects had influenza virus.

**FIGURE 2:**
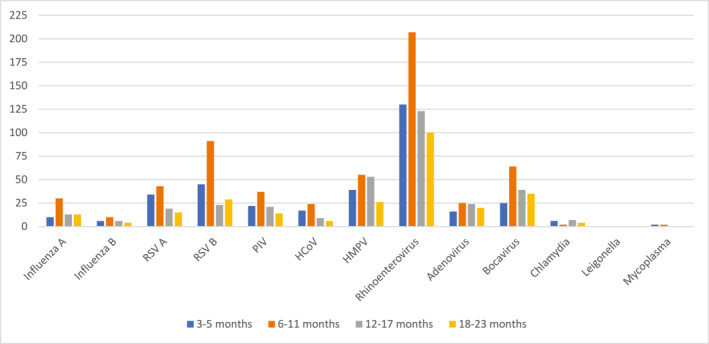
Distribution of respiratory viruses by age, rural Bangladesh 2016–2017. PIV includes PIV1, PIV2, PIV3, and PIV4; HCoV includes HCoV 229E, HCoV NL63, HCoV OC43, and HCoV HKU1.

Overall, HRV/HEV was the most frequently identified virus (37.9%), followed by RSV (20.2%) and hMPV (11.7%). Among RSV, subtype B was more common than subtype A. The viruses most commonly involved in dual virus isolation were HRV/HEV (*n* = 165) and bocavirus (*n* = 99) (Table 3). Across all age group categories as shown in Figure 2, HRV/HEV was the most common virus detected, followed by RSV and hMPV.

**FIGURE 3:**
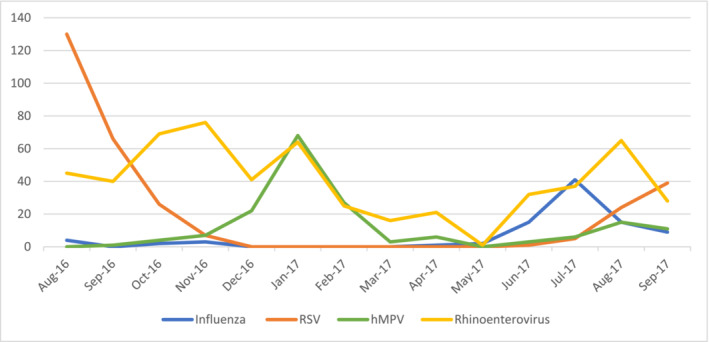
Proportion of children aged 3–23 months with viral ALRI by month, rural Bangladesh, 2016–2017.

The proportion of ALRI in which a respiratory virus was detected was highest during the autumn season (mid‐June to mid‐October). Specifically, influenza virus detection was recovered more frequently in those who presented during the months of June–August, RSV during August–November, and hMPV during December–March, following the RSV peak. For HRV/HEV, detection was year‐round, but HRV/HEV was identified more often in children enrolled during the months of November 2016 and August 2017 (Figure 3).

Table 4 shows the clinical characteristics of children from whom specific viruses were recovered. History of fever was less common in those from whom HRV/HEV was recovered (Table 4); however, a large proportion of those with HRV/HEV presented with a history of cough (96.2%), age‐adjusted fast breathing (72.3%), chest indrawing (82.3%), and crepitations on lung auscultation (67.5%). Fever was most frequently noted among those infected with influenza virus (83.7%), and respiratory symptoms (such as fast breathing, chest indrawing and nasal flaring) were most common in those infected with RSV. Chest radiograph findings of alveolar consolidation with or without other infiltrates were greatest among children infected with hMPV (34.0%), followed by those infected with influenza virus. The need for in‐patient hospitalization was greatest among those infected with influenza virus (29.3%), followed by those infected with RSV (26.1%) (Table 4).

**TABLE 4:** Clinical characteristics of children with specific respiratory viruses.

Characteristics^a^	Influenza A + B (*n* = 92)^b^	RSV A + B (*n* = 299)^b^	PIV (*n* = 96)^b^	HMPV (*n* = 173)^b^	HRV/HEV (*n* = 560)^b^
Sex, male	54 (58.6)	164 (55.0)	56 (58.3)	91 (52.6)	344 (61.4)
Median age in months (SD)	10 (9.5)	9 (8)	10 (8)	10 (9)	10 (9)
**Reported symptoms (last 3 days)**					
History of fever	81 (88.0)	238 (79.8)	76 (79.1)	141 (81.5)	371 (66.2)
History of cough	86 (93.4)	281 (94.2)	93 (96.8)	165 (95.3)	539 (96.2)
History of difficulty breathing	55 (59.7)	197 (66.1)	63 (65.6)	115 (66.4)	381 (68.0)
History of runny nose	55 (59.7)	92 (30.8)	50 (52.0)	87 (50.2)	247 (44.1)
**Degree of fever**					
≥ 101°F	47 (16.3)	125 (41.8)	30 (31.3)	74 (42.8)	137 (24.5)
99.5°F–100.9°F	11 (11.9)	55 (18.3)	24 (25.0)	24 (13.9)	82 (14.6)
98.6°F–99.4°F	19 (20.6)	59 (19.7)	19 (19.8)	30 (17.3)	140 (25.0)
Afebrile	15 (16.3)	60 (20.0)	23 (23.9)	45 (26.0)	201 (35.9)
Observed cough	39 (42.3)	89 (29.8)	33 (34.3)	62 (35.8)	169 (30.1)
Fast breathing	58 (63.0)	227 (76.1)	73 (76.0)	128 (73.9)	405 (72.3)
Chest indrawing	64 (69.5)	247 (82.8)	69 (71.8)	140 (80.9)	461 (82.3)
Crepitations	44 (47.8)	230 (77.1)	64 (66.6)	136 (78.6)	378 (67.5)
Wheeze on auscultation	21 (22.8)	85 (28.5)	22 (22.9)	38 (21.9)	170 (30.3)
Audible wheeze	1 (1.0)	0 (0.0)	0 (0.0)	1 (0.5)	1 (0.0)
**Hypoxemia (< 90% sat)**	3 (3.2)	19 (6.3)	6 (6.2)	8 (4.6)	25 (4.4)
94–100	64 (69.6)	229 (76.8)	72 (75.0)	141 (81.5)	441 (78.8)
90–93	3 (3.2)	35 (11.8)	6 (6.2)	12 (6.9)	47 (8.4)
< 90	3 (3.2)	19 (6.4)	6 (6.2)	8 (4.6)	25 (4.4)
Missing	22 (23.9)	15 (5.0)	12 (12.5)	12 (6.9)	47 (8.3)
Head nodding or tracheal tugging	4 (4.3)	49 (16.4)	6 (6.2)	20 (11.5)	66 (11.7)
Nasal flaring	7 (7.6)	59 (19.7)	5 (5.2)	11 (6.3)	46 (8.2)
Grunting	0 (0.0)	2 (0.0)	0 (0.0)	2 (1.1)	1 (0.0)
**General danger sign**					
Convulsions	6 (6.5)	6 (2.0)	0 (0.0)	2 (1.1)	11 (1.9)
Lethargy	0 (0.0)	0 (0.0)	0 (0.0)	1 (0.5)	3 (0.0)
Unable to eat or drink	0 (0.0)	0 (0.0)	0 (0.0)	0 (0.0)	0 (0.0)
Vomits everything	0 (0.0)	0 (0.0)	0 (0.0)	0 (0.0)	0 (0.0)
**Chest radiography**					
Alveolar consolidation only	2 (2.1)	7 (2.3)	5 (5.2)	10 (5.7)	16 (2.8)
Other infiltrate only	21 (22.8)	49 (16.3)	12 (12.5)	39 (22.5)	93 (16.6)
Alveolar consolidation & other infiltrate	23 (25.0)	56 (18.7)	17 (17.7)	49 (28.3)	109 (19.4)
**Hospitalized**	27 (29.3)	78 (26.1)	19 (19.7)	34 (19.6)	92 (16.4)
**Duration of hospitalization**					
Median (SD) in days	2 (7.4)	2 (0.8)	2 (1.2)	2 (1.2)	2 (1.4)
< 1 day	4 (14.8)	1 (1.2)	4 (21.0)	1 (2.9)	8 (8.6)
1–2 days	17 (62.9)	64 (82.0)	10 (52.6)	21 (61.7)	61 (66.3)
3–4 days	5 (18.5)	12 (15.3)	5 (26.3)	10 (29.4)	18 (19.5)
≥ 5 days	1 (3.7)	1 (1.2)	0 (0.0)	2 (5.8)	5 (5.4)
**Outcome of hospitalization**					
Discharged	25 (92.5)	78 (100.0)	19 (100.0)	33 (97.0)	88 (95.6)
Referred	1 (3.7)	0 (0.0)	0 (0.0)	1 (3.0)	1 (1.0)
Left against medical advice	1 (3.7)	0 (0.0)	0 (0.0)	0 (0.0)	2 (2.1)
Died	0 (0.0)	0 (0.0)	0 (0.0)	0 (0.0)	1 (1.0)

^a^ Percentages are all column percentages, unless indicated otherwise. Percentages are rounded off to the first decimal point.

^b^ Analyses describe cases where specific virus was detected, irrespective of single or multiple virus detection.

## 4 Discussion

This study provides data on viral respiratory pathogens associated with ALRI in children 3–23 months of age attending three surveillance hospitals in rural Sylhet district of Bangladesh. We detected respiratory viruses in a high proportion of children (81.8%); the top four viruses identified were HRV/HEV, RSV, hMPV, and influenza virus. Viruses were detected disproportionately in children with ALRI presenting during the autumn season, and influenza virus and RSV were most frequently associated with the need for in‐patient hospitalization.

The preponderance of virus associated‐severe ALRI in our study was noteworthy, and in keeping with findings from the Bangladesh arm of the PERCH study, where 77.7% of severe ALRI (referred to as severe or very severe pneumonia in the PERCH study defined using the modified 2005 WHO criteria) cases were attributed to respiratory viruses [10]. Similar proportions of respiratory viral etiology were found in urban Bangladesh, both in community‐based [20] and hospital‐based [21] ALRI cohorts. Other studies examining respiratory viral etiology for ALRI among children in Europe, Africa, east Asia, and southeast Asia have found similar isolation rates [22, 23, 24]. These findings are important for both epidemiologists as well as clinicians to consider, as in many settings, antibiotics are used to treat ALRIs, although the etiology may not necessarily be bacterial. Our findings call for wider availability and inclusion of testing for respiratory viruses to better understand the etiology and treatment options for pediatric ALRI.

In our study, the most commonly identified viruses were HRV/HEV, RSV, and hMPV. Although rhinoenteroviruses have been historically thought to cause mild self‐limiting upper respiratory infection, they are increasingly being identified as causes of severe bronchiolitis, and severe ALRI, including radiograph‐confirmed pneumonia [25], and are associated with long‐term sequelae including recurrent wheezing and the development of asthma in young children [26, 27]. The PERCH study, which rigorously assessed bacterial and viral etiology among children aged 1–59 months hospitalized with severe or very severe ALRI at multiple sites across the globe and included controls found that Bangladesh had the greatest prevalence of HRV/HEV across the study sites, and this is likely attributable to the greatest population density and crowding of any country [10]. The high prevalence of undernutrition could also be contributory, as was found in other settings. [28]. The PERCH study (which also included asymptomatic controls) and other Bangladeshi studies [20, 29] have also found RSV to be an important etiological agent for childhood ALRI. As observed in our study, in temperate climate regions, RSV circulation is seen typically during fall and winter [30]. RSV frequently causes severe ALRI that requires hospitalization in young children [31] and is associated with a high burden of morbidity and mortality especially among children < 2 years [32]. The recent development of an array of monoclonal antibodies as well as vaccines for RSV prevention are promising but ensuring that these interventions are financially feasible and widely available in resource‐limited regions remains a critical hurdle. Overcoming these challenges will require concerted efforts from the global health community, fostering collaborations that prioritize equitable access and affordability.

HMPV was the third most common virus detected in this study. Previous studies have reported that hMPV is prevalent during late winter and spring in temperate climates [33], and our findings were similar. The virus is highly contagious and spreads through respiratory droplets, making it a frequent cause of respiratory infections in childcare and community settings. In young children, especially those with underlying health conditions, hMPV infections can lead to severe respiratory distress and hospitalization [33]. Understanding the role of hMPV as a cause of ALRI in children less than 2 years of age is crucial for designing targeted preventive strategies, including potential vaccines and therapeutic interventions, to mitigate the impact of this respiratory virus on pediatric health. Our findings that hMPV infections peak after the RSV peak also have important implications for future vaccine programs, especially because vaccines targeting both viruses together are currently in early‐phase clinical trials [34, 35].

We found that almost one‐fourth of children with ALRI had two or more respiratory viruses detected, involving most commonly HRV/HEV, bocavirus, and hMPV. Similar rates of viral co‐infection have been reported in other studies from the region [10, 36]. In a systematic review, Goka et al. reported that the incidence of mixed viral infections ranged from 5%–62%, with a mean of 23% [37]. Although some studies have suggested that multiple infections were associated with increased morbidity and mortality, this finding is not conclusive [37]. Furthermore, the simultaneous presence of more than one virus in the same sample must be interpreted cautiously. Highly sensitive PCR assays may identify very small amounts of viral nucleic acids present during the incubation period or the convalescence phase of the illness. The identification of more than one virus can also be explained by the prolonged shedding of the virus that caused a previous infection, a coincidental upper airway infection, or the asymptomatic circulation of some viruses [38]. Bocavirus is frequently found in the presence of other respiratory viruses, making it difficult to establish a direct causal relationship with ALRI. A study of viral etiology for ALRI among hospitalized children from the Sa Kaeo Province in Thailand found that 91% of bocavirus‐positive ALRI patients < 5 years of age were co‐infected with another virus [39] and prolonged bocavirus shedding has been reported for up to 4.5 months in hospitalized children [40], making the role of bocavirus as a pathogen unclear. In contrast, RSV, influenza, hMPV, and PIV are significantly more frequent in patients with infection than in controls, and their detection by PCR is likely to be causal [41, 42]. This study was conducted prior to the emergence of the SARS‐CoV‐2 pandemic. Consequently, the data presented here may not reflect the current landscape of viral pathogens in the causation of ALRI in young children. In our study, the seasonal coronaviruses (coronavirus types NL63, HKU1, 229E, and OC43) were not frequent agents associated with ALRI, a finding similar to those reported in other studies [10, 43, 44]. Future research will help elucidate how the introduction of the novel SARS‐CoV‐2 could change the occurrence and interaction of respiratory pathogens globally and better define the role SARS‐CoV‐2 will play in the causation of pediatric ALRI the postpandemic era.

This study has several limitations. First, only a small fraction of children with ALRI from the study area sought care from study hospitals [12]. Second, we did not have data regarding bacterial etiologies, so we could not assess bacterial‐viral co‐infection. Third, we did not have control data, so we could not attribute causality to viruses that are frequently associated with colonization. In some cases, certain viruses not associated with ALRI may have been detected; however, the true etiology could have been bacterial. Fourth, a large number of children (28.5%) could not be tested for viral etiology; however, there were no major differences in the baseline demographics between those tested for respiratory viruses and those who could not be tested. Despite these limitations, this study is among the first to describe the epidemiology and clinical features of respiratory viral‐associated ALRI in rural Bangladesh and provides important insights into future studies and subsequent prevention strategies.

In conclusion, we identified respiratory viruses in a large proportion of children < 2 years presenting to care with ALRI in rural Bangladesh. The data on seasonality of respiratory viruses and associated ALRI can be valuable for determining the timing of administration of respiratory vaccines, for example, providing influenza vaccines prior to the onset of the influenza season in the monsoon and autumn seasons. Our findings suggest that further studies in which rigorous confirmation of respiratory viral agents is performed in children with ALRI are necessary, particularly as prevention strategies such as vaccine programs and antimicrobial stewardship programs are implemented.

## Author Contributions

Funding acquisition: AHB and MER. Conceptualization and design: MER, AHB, EDM, LHM, SA, SS, and MS. Data curation: MER, NHC, ADR, LHM, and AHB. Data collection: ADR, JA, and NHC. Data analysis: MER, KM, NHC, and AHB. Data interpretation: MER, KM, ADR, EDM, SA, LHM, and AHB. Writing – original draft: MER and KM. Writing – review and editing: EDM, SA, NHC, ADR, MS, LHM, SS, and AHB.

## Ethics Statement

The National Research Ethics Committee of Bangladesh Medical Research Council and the institutional review boards of the Johns Hopkins and Duke Schools of Medicine reviewed and approved the study's protocol.

## Conflicts of Interest

The authors declare no conflicts of interest.

### Peer Review

The peer review history for this article is available at https://www.webofscience.com/api/gateway/wos/peer‐review/10.1111/irv.70062.

## Data Availability

All relevant data are available within the paper tables and figure files.
